# Association Between Workplace Gaslighting and Perceived Quality of Care, Patient Safety and Quiet Quitting: A Cross-Sectional Study Among Nurses in Greece

**DOI:** 10.3390/healthcare14040450

**Published:** 2026-02-11

**Authors:** Ioannis Moisoglou, Aglaia Katsiroumpa, Ioanna V. Papathanasiou, Olympia Konstantakopoulou, Aris Yfantis, Angeliki Katsapi, Petros Galanis

**Affiliations:** 1 Department of Nursing, University of Thessaly, 41500 Larissa, Greece; iopapathanasiou@uth.gr; 2 Clinical Epidemiology Laboratory, Faculty of Nursing, National and Kapodistrian University of Athens, 11527 Athens, Greece; aglaiakat@nurs.uoa.gr (A.K.); pegalan@nurs.uoa.gr (P.G.); 3 Center for Health Services Management and Evaluation, Faculty of Nursing, National and Kapodistrian University of Athens, 11527 Athens, Greece; olympiak1982@hotmail.com; 4 Special Vocational Education Laboratory of Fthiotis, Directorate of Secondary Education of Fthiotis, 35100 Lamia, Greece; arisyfantis@gmail.com; 5 Euro-Mediterranean Institute for Quality and Safety in Health Services, 10678 Athens, Greece; akatsapi@eiqsh.eu

**Keywords:** gaslighting, nurses, patients, quality of health care, safety, workplace

## Abstract

**Background:** Workplace gaslighting, as a form of psychological manipulation, may negatively affect nurses’ work behaviors and perception of care. However, its connection to perceived quality of care, patient safety and quiet quitting has not been sufficiently explored. **Objectives:** To examine the impact of workplace gaslighting on perceived quality of care, patient safety and quiet quitting in nurses. **Methods:** A cross-sectional study with a convenience sample was conducted in Greece. We used the Gaslighting at Work Scale and the Quiet Quitting Scale to measure workplace gaslighting and quiet quitting, respectively. We used IBM SPSS 28.0 to perform logistic regression analysis and linear regression analysis. Significance level was set at 0.05. **Results:** Mean age of nurses was 42.98 years, while females comprised 82.1% of them. More than half of our nurses (52.0%) evaluated the quality of care in their unit as good, while 33.1% perceived patient safety as good. A higher level of workplace gaslighting was significantly associated with lower odds of reporting perceived quality of care to be good or excellent. Increased workplace gaslighting was also associated with decreased odds of good-to-excellent patient safety. Moreover, workplace gaslighting was significantly and positively associated with quiet quitting. **Conclusions:** Our study supports the negative impact of workplace gaslighting on perceived quality of care, patient safety and quiet quitting. Establishment of clear policies and procedures that encourage staff to report such behaviors, is essential to dismantle the barriers created by psychological manipulation.

## 1. Introduction

Nursing leadership, the manner in which an individual influences nurses’ attitudes and behaviors, can also determine the effectiveness of the nursing care provided. The leadership style most consistently identified as pivotal to fostering a safety culture among nursing staff is transformational leadership [1,2]. Core attributes of transformational leadership, such as leveraging errors as opportunities for learning and improvement, establishing a blameless safety culture, promoting open multidisciplinary communication, and actively involving followers in decision making, constitute fundamental elements for cultivating a robust culture of safety [3]. In contrast to transformational leadership, toxic leadership represents a style that effectively undermines any effort to develop and sustain a safety culture [4]. Specifically, toxic behavior may encompass features such as narcissistic behavior, referring to patterns of extreme self-centeredness and an inflated sense of personal importance; self-promoting behavior, involving actions aimed at advancing one’s own interests, such as exploiting staff and exhibiting marked shifts in conduct when interacting with superiors; and humiliating behavior, which includes practices that shame or embarrass employees, demonstrate limited concern for personnel or the organization, and reflect a lack of respect or consideration, including disparaging feedback, inequitable treatment, and the imposition of excessive pressure on nursing staff [4].

Gaslighting is a behavioral pattern that closely resembles toxic conduct, functioning as a form of psychological manipulation and a manifestation of structural power. In the introduction to the second edition of her book, Stern, an author and psychoanalyst, proposes the following definition of gaslighting: “*Gaslighting, is a type of emotional manipulation in which a gaslighter tries to convince you that you’re misremembering, misunderstanding, or misinterpreting your own behaviors or motivations, thus creating doubt in your mind that leaves you vulnerable or confused*” [5]. Its core features are inherently misaligned with the prerequisites for cultivating a robust safety culture. In practice, perpetrators may fabricate information, undermine the target’s perceptions and recollections, question emotional or cognitive responses, and/or manipulate contextual cues in ways that generate disorientation and a sense of unreality. Taken together, these tactics aim to destabilize the individual’s confidence in their own judgment and affect, fostering self-doubt and progressively diminishing self-trust and self-esteem. Notably, gaslighters often repudiate objective facts even when confronted with credible, well-substantiated evidence. This pattern of behavior is commonly rooted in motives of dominance and control, reinforced by personal insecurity, a compulsive need for correctness, and a drive for power [6]. Employees, encompassing nurses, who are victims of gaslighting, report higher levels of occupational burnout, greater turnover intention and quiet quitting, and lower work engagement while also facing serious mental health issues, including anxiety and depression [7,8]. Gaslighting behaviors foster a work environment that acts as a barrier to the development of a patient safety culture.

A work-related behavior that first gained prominence in the business sector during the COVID-19 pandemic is quiet quitting [9]. In an effort to push back against a culture of relentless striving, often in the absence of meaningful organizational attention to employee well-being, and to achieve a better balance between work and personal life, employees may adopt quiet quitting. In practice, this involves deliberately scaling back discretionary effort, limiting performance to the minimum requirements of the role, refraining from going above and beyond, and focusing primarily on meeting the formal job description [9]. In the healthcare sector, an increasing number of health professionals, most notably nurses, are opting for quiet quitting [10]. Within nurses’ exceptionally challenging work environment, often characterized as poor, with very high workloads and elevated turnover intention [11,12,13], factors that collectively contribute to the emergence of quiet quitting, this phenomenon appears to represent the Achilles’ heel of health systems. Specifically, reduced discretionary effort may undermine any sustained attempt to continuously improve the quality of nursing care delivery.

Patient safety constitutes the most critical dimension of the quality of healthcare services and has become a central priority for the leadership of healthcare organizations worldwide. The publication of the Institute of Medicine (US) report “To Err is Human: Building a Safer Health System” profoundly shook health systems worldwide, as it revealed that nearly 100,000 patients were dying each year in U.S. hospitals, not due to their underlying disease, but as a result of errors occurring during the delivery of care [14]. At the same time, this report catalyzed a systematic, concerted effort to reduce errors that lead to adverse patient events. It is estimated that 10–12% of hospitalized patients experience some form of adverse event, including healthcare-associated infections, medication errors, pressure ulcers, procedure-related complications, and falls. Adverse events are not an issue confined to the United States; rather, they have a substantial global impact, placing a significant burden on healthcare systems across all continents, with the proportion of patients experiencing at least one adverse event ranging from 7.3% to 21.9% [15].

The consequences of adverse events for patients and healthcare organizations include emotional and physical harm, such as pain and disability, with a proportion resulting in patient death, as well as prolonged length of hospital stay, increased likelihood of readmission and increased costs of care [15,16,17,18]. The multidimensional consequences of adverse events also affect healthcare professionals, who may experience second victim syndrome, manifested through a wide range of symptoms including troubling memories, anxiety and concern, self-directed anger, regret and remorse, distress, fear of future errors, embarrassment, guilt, reduced self-confidence, and sleep disturbances [19]. Approximately one in five healthcare professionals requires up to one year to recover, and in some cases they may never fully recover [20]. Despite sustained efforts by healthcare organizations’ leadership and a reduction in the incidence of adverse events, these events continue to pose a threat to patients’ health status and lives, substantially undermining the quality of healthcare services [21].

The quality of care delivered is not, of course, confined to patient safety; rather, it is a multidimensional construct that health professionals conceptualize as holistic care. From nurses’ perspectives, care quality may encompass domains such as effective communication, teamwork, optimal patient outcomes, competence, knowledge, satisfaction, and meeting patients’ needs [22]. These needs may include treating patients with respect and dignity; acknowledging and supporting their spiritual, cultural, religious, and sexual identity; supporting patients in making informed choices; effective pain management; adequate patient monitoring/surveillance; educating patients and/or family members; and preparing patients and families for discharge [23,24].

To date, the literature examining the relationship between leadership and the quality and safety of patient care has primarily focused on the positive effects of specific leadership models (e.g., transformational leadership), as well as on the detrimental impact of toxic leadership. Although gaslighting has been extensively studied within sociological and psychological frameworks, particularly in the context of interpersonal relationships, there remains a notable gap in the literature regarding its prevalence in the healthcare workplace and its impact on the quality and safety of nursing care delivery.

To the best of our knowledge, the present study is the first to examine the impact of workplace gaslighting on perceived quality of care, patient safety and quiet quitting on nursing staff.

## 2. Materials and Methods

### 2.1. Study Design

A cross-sectional study was conducted in Greece, and data were collected using an online survey from October to November 2025. The questionnaire was administered via Google Forms, chosen for its accessibility and compatibility with multiple digital devices. To facilitate broad dissemination, the survey link was shared through nurses’ professional social media groups on Facebook, Instagram and LinkedIn. Nurses’ networks represented institutional and professional association networks of nurses, enabling efficient outreach to practicing nurses across various healthcare settings. This process yielded a convenience sample, as participation depended on voluntary engagement through these digital channels.

Eligible participants were required to meet specific inclusion criteria. First, individuals had to be currently employed as clinical nurses working in hospitals. Second, they had to occupy subordinate clinical roles, meaning they were not supervisors or managers of other nurses. Third, participants were required to have at least one year of work experience in their current nursing position to ensure adequate exposure to clinical routines and workplace environments. Finally, all nurses were required to provide informed consent electronically before accessing and completing the study questionnaire. Only participants who met all inclusion criteria and consented to participate were included in the final sample.

This study adhered to the Strengthening the Reporting of Observational studies in Epidemiology (STROBE) guideline [25].

We used G*Power v.3.1.9.2 for sample size determination. Sample size determination was conducted a priori. The calculation was based on the analytical requirements of our multivariable regression models, which included five variables (gender, age, years of work experience, working in shifts and working in an understaffed department) considered potential confounders in the association between our predictor (i.e., workplace gaslighting) and our study outcomes (perceived quality of care, perceived patient safety, quiet quitting). We assumed an effect size of 0.03 between our predictor and outcomes, corresponding to a small effect. To ensure robust statistical inference, the analysis was set at a high desired statistical power of 95% (1 − β = 0.95), thereby reducing the risk of type II error. The significance level (α) was set at the conventional threshold of 0.05. Under these specifications, the power analysis indicated that a minimum sample size of 436 nurses was required to reliably detect the expected effect size after adjusting for the five confounding variables.

### 2.2. Measurements

Demographic data included: gender (male/female), age (continuous variable), years of work experience (continuous variable), working in shifts (yes/no), and working in an understaffed department (yes/no).

The Gaslighting at Work Scale (GWS) was used to evaluate workplace gaslighting among nurses [26]. It consists of two factors: “loss of self-trust” (five items) and “abuse of power” (six items). The items are rated on a 5-point Likert scale ranging from 1 (never) to 5 (always). Total and subscale scores were calculated as an average of all responses (1–5), with higher scores indicating more frequent behaviors of supervisor gaslighting. In particular, we added the answers to the 11 items, and we divided the sum by 11. Also, to calculate “loss of self-trust” score, we added the answers to the five items, and we divided the sum by five, while, to calculate “abuse of power” score, we added the answers to six items, and we divided the sum by six. The developers of the scale recommend an optimal cut-off point of 2.1. Employees with a total GWS score greater than or equal to this value are classified as experiencing workplace gaslighting, while those with a total score below the cut-off point are considered not to exhibit workplace gaslighting. Several aspects of validity of GWS have already been demonstrated, including content validity, face validity, construct validity and concurrent validity. The GWS version in the Greek language was used [27], yielding a Cronbach’s alpha value of 0.948. The Cronbach’s alpha values for “loss of self-trust” and “abuse of power” were 0.924 and 0.913, respectively.

To assess quiet quitting among nurses, we used the 9-item Quiet Quitting Scale (QQS) [28]. Answers were recorded on a 5-point Likert scale from one (strongly disagree/never) to five (strongly agree/always). It consists of three factors: “detachment” (four items), “lack of initiative” (three items) and “lack of motivation” (two items). Score for each factor was derived by calculating the average value of the responses to items, ranging from 1 to 5. Similarly, the total score on the QQS is calculated as the mean of the nine items. Specifically, the responses to all nine items are summed, and the resulting total is then divided by nine. Higher scores are associated with higher levels of quiet quitting. The validated version of the QQS in Greek was used [10]. Multiple forms of evidence supporting the validity of the QQS have already been established, including its content, face, construct, and concurrent validity. In our study, Cronbach’s alpha for the QQS was 0.814, for the factor “detachment”, it was 0.737, for the factor “lack of initiative”, it was 0.693, and for the factor “lack of motivation”, it was 0.810.

Perceived quality of care and perceived patient safety were measured with single items, representing the overall assessments in nurses’ unit. Quality of care was assessed with the question: “How would you rate the quality of the nursing care provided in your unit?” with answers in a 4-point Likert scale; poor, fair, good and excellent. This question constitutes a reliable approach for assessing the quality of nursing care delivery and is used internationally [29].

Patient safety was measured by the following question: “How would you assess patient safety in your unit?” with answers in a 5-point Likert scale; poor, fair, good, very good and excellent. This item was dichotomized for analysis (0 = poor/fair and 1 = good/very good/excellent) as the literature suggests [30]. Increased values reflect increased ratings for perceived quality of care and patient safety.

### 2.3. Ethical Issues

Our study was conducted in accordance with the guidelines of the Declaration of Helsinki [31]. Our study protocol was approved by the Ethics Committee of the Faculty of Nursing, National and Kapodistrian University of Athens (approval number: 08, 23 September 2025). Data was collected in an anonymous and voluntary manner. Participants were informed about the purpose and design of the study and provided researchers with their consent.

### 2.4. Statistical Analysis

We use mean, standard deviation (SD), median, and interquartile range to present continuous variables. Also, we use numbers and percentages to present categorical variables. Continuous variables followed the normal distribution. Workplace gaslighting was the predictor, and perceived quality of care, perceived patient safety and quiet quitting were the outcomes. Demographic variables (gender, age, years of work experience, working in shifts, and working in an understaffed department) were considered as confounding factors.

Logistic regression analysis was used to explore associations between workplace gaslighting, perceived quality of care and perceived patient safety. Final multivariable logistic regression models were built to determine the independent effect of workplace gaslighting, after adjustment for confounding. Age was highly correlated with years of work experience (Pearson’s correlation coefficient = 0.912, *p* < 0.001) suggesting multicollinearity issues; thus, we added one of these two variables into the final multivariable models (work experience instead of age). The findings are reported as crude and adjusted odds ratios (ORs) with 95% confidence intervals (CIs) and *p*-values.

Linear regression analysis was performed to detect the association between workplace gaslighting and quiet quitting. We finally built a multivariable model controlled for the confounders we mentioned above. We present crude and adjusted betas, 95% CI, and *p*-values. Additionally, we evaluated the assumptions of multivariable linear regression models, such as multicollinearity, multivariable normality, homoscedasticity and linearity [32]. *p*-values less than 0.05 were considered statistically significant. We performed statistical analyses with IBM SPSS 28.0 (IBM Corp. Released 2021. IBM SPSS Statistics for Windows, Version 28.0. Armonk, NY, USA: IBM Corp.).

## 3. Results

### 3.1. Demographic Characteristics

Participants’ demographic and work-related characteristics are presented in Table 1 (*n* = 492). Females comprised 82.1% of respondents, the average age of nurses was 42.98 years (SD = 18.27), and the mean duration of work experience was 9.79 years (SD = 9.95). Most of the participants were involved in shift work (81.7%) and had been working at an understaffed department (85.2%).

### 3.2. Study Scales

A mean score of 1.78 (SD = 0.89) was reported on the Gaslighting at Work Scale, while abuse of power scored higher (mean = 1.93, SD = 1.00) than loss of self-trust (mean value = 1.59, SD = 0.86).

Quiet quitting levels were similarly low to moderate (mean value = 2.18, SD = 0.65). Lack of motivation had the highest mean score (mean value= 2.77, SD = 1.00), followed by lack of initiative (mean value = 2.12, SD = 0.84) and detachment (mean value = 1.93, SD = 0.73), implying that motivational disengagement was the most emphasized, in contrast to behavioral withdrawal or emotional detachment.

In our sample, 24.4% of nurses suffered from workplace gaslighting (gaslightees) according to the GWS score. Moreover, more than half of our nurses (55.3%) were considered quiet quitters according to their score on QQS. Status of the study scales is presented in Table 2.

Table 3 presents the Pearson correlation coefficients between study scales and their subscales. Workplace gaslighting was significantly and positively associated with the QQS. Similarly, all workplace gaslighting subscales were significantly and positively associated with all quiet quitting sub-scales. These findings indicate that higher perceived workplace gaslighting was correlated with greater levels of quiet quitting.

### 3.3. Quality of Care and Patient Safety

Almost half of the participants (52.0%, *n* = 256) evaluated the quality of care in their unit as good, while 23.6% (*n* = 116) evaluated it as fair, 19.7% (*n* = 97) as excellent and 4.7% as (*n* = 4.7%) poor. Thus, five out of ten nurses considered the quality of care in their unit as good, and two out of ten nurses considered the quality of care as excellent.

Moreover, 33.1% (*n* = 163) of nurses perceived patient safety as good, 28.5% (*n* = 140) as very good, 20.7% (*n* = 102) as fair, 11.2% (*n* = 55) as excellent and 6.5% (*n* = 32) as poor. Therefore, 61.6% of nurses perceived patient safety as good/very good.

### 3.4. Association Between Workplace Gaslighting and Perceived Quality of Care and Patient Safety

Table 4 presents the unadjusted and adjusted effects of workplace gaslighting on perceived quality of care and patient safety among nurses, based on univariate and multivariable logistic regression analyses.

In the univariate comparisons, greater workplace gaslighting was significantly associated with lower odds of reporting perceived quality of care to be good or excellent. This association was still statistically significant in the multivariable model after gender, years of work experience, working in shifts and working in an understaffed department were included.

Workplace gaslighting was also strongly related to perceived patient safety. In the univariate analysis, increased workplace gaslighting was associated with decreased odds of good-to-excellent patient safety. This association remained after controlling for the potential confounders.

These results suggest that higher perceived workplace gaslighting was related to worse perceptions of quality care and patient safety, even after adjusting for key demographic/work-related variables.

### 3.5. Association Between Workplace Gaslighting and Quiet Quitting

Univariate and multivariable linear regression analyses for workplace gaslighting and quiet quitting are shown in Table 5. In the univariate analysis, workplace gaslighting was significantly and positively associated with quiet quitting, indicating that higher levels of workplace gaslighting were related to increased quiet quitting behaviors. This association was still significant even when gender, years of work experience, working in shifts and working in an understaffed department were considered. These results demonstrate that exposure to workplace gaslighting was independently related to greater tendency toward quiet quitting among nurses beyond demographic and work-related covariates. The multivariable model explained 13% of the variation in quiet quitting (R^2^ = 13.0%) and was statistically significant (ANOVA *p*-value < 0.001). Multivariable regression model assumptions were met (see Figures S1 and S2 in the supplementary material, while VIFs ranged from 1.006 to 1.247).

## 4. Discussion

The present study is the first to investigate and highlight the significant association between workplace gaslighting and the quality and safety of care, as well as nurses’ quiet quitting. Given the existing gap in the literature, the findings will be discussed in the context of gaslighting’s impact on nurses’ work-related behavior, which has, in turn, been empirically shown to influence the quality and safety of nursing care delivery.

Our finding regarding the effect of gaslighting on nurses’ quiet quitting is consistent with evidence from studies on employees outside the healthcare sector, where gaslighting has been associated not only with quiet quitting but also with reduced work engagement and poorer mental health, including increased anxiety and depressive symptoms [8]. Quiet quitting among nurses often constitutes a precursor to their departure from the employing organization and, in some cases, from the profession altogether [11]. As nurses’ turnover intention increases, the quality and safety of nursing care correspondingly decline [33,34]. Even when nurses wish to advance their careers and leave their current career trajectory, gaslighting constitutes a substantial barrier. Remaining “trapped” in a specific role and organization may also adversely affect the quality of care delivered [35]. Furthermore, gaslighting deprives nurses of the capacity to adapt rapidly and effectively to the continuously evolving healthcare environment [36], an adaptive capability through which they can enhance the safety of care [37]. Nurses who are subjected to gaslighting by their supervisors are more likely to develop occupational burnout [7]. Patients hospitalized in units where nurses experience burnout may be at increased risk of adverse events and report lower satisfaction with the care received; concurrently, burnout has been associated with lower nurse-assessed quality of care [38].

The results of the present study revealed that higher perceived workplace gaslighting was related to worse perceptions of quality care and patient safety, even after adjusting for key demographic/work-related variables. Since this study is the first to examine the impact of gaslighting on the quality and safety of nursing care, the aforementioned finding will be discussed within the broader context of leadership’s overall influence on the quality and safety of care. When models of toxic nursing leadership are implemented, healthcare organizations report increased adverse outcomes, particularly a higher incidence of hospital-acquired infections, patient falls, medication errors, and complaints from patients’ family members, compared with work environments in which transformational, authentic, and inclusive leadership are practiced [39]. When nurse managers adopt inclusive leadership, they enhance nurses’ intention to report errors [39]. Error reporting constitutes a fundamental prerequisite for investigating the factors that contribute to errors and, consequently, for improving the safety of care delivery. The characteristics of authentic leadership, which emphasize the development of a healthy workplace environment and focus on core leader qualities, such as self-awareness, relational transparency, an internalized moral perspective, and balanced processing, are associated with a decrease in nurse-assessed adverse events and an increase in the quality of care [40].

In contrast to gaslighting behaviors, where employees may be driven to doubt their own judgment, be portrayed as “crazy” when they voice their views, and consequently become stigmatized and socially isolated, fostering a safety culture requires leadership that encourages nurses to speak up, identify patient safety issues, address them effectively, and provide timely feedback within a learning-oriented environment that leverages errors as opportunities for improvement and cultivates psychological safety [41,42]. Such leadership is non-punitive, demonstrates trust in staff, and actively promotes collaboration across the team. Both nurse-to-nurse communication and interprofessional communication constitute essential prerequisites for ensuring patient safety [43,44]. Moreover, nurse managers who adopt these leadership practices enhance nurses’ work engagement and reduce burnout, thereby creating conditions conducive to fewer errors and adverse events [41,42].

The present study has several limitations. First, its cross-sectional design precludes the establishment of causal relationships among the variables examined. In addition, the assessment of care quality and safety relied on self-reported measures, and no administrative data from patient records were used. Therefore, participants’ responses may have been influenced by subjective appraisal and reporting bias. Since we used a convenience sample of nurses to examine the association between workplace gaslighting, quality of care, patient safety and quiet quitting, we cannot generalize our findings to the population of nurses in Greece or other countries. Further studies with random, stratified and more representative samples of nurses should be conducted to further validate our findings. Finally, to our knowledge, this is the first study to investigate the association between workplace gaslighting and the quality and safety of care. Further studies in other countries are warranted to corroborate the present findings.

## 5. Conclusions

The quality and safety of nursing care delivery are central priorities across health systems worldwide. The consequences of adverse events are exceptionally serious and multifaceted, affecting patients, healthcare organizations, and health professionals alike. Although nurses working under demanding conditions require strong managerial support, they often instead become targets of abusive behaviors such as gaslighting, which undermines care quality and safety and may contribute to nurses’ quiet quitting. The present study is the first to highlight the significant association between workplace gaslighting and the quality and safety of care, as well as nurses’ quiet quitting. A zero-tolerance stance by senior leadership, coupled with the establishment of clear policies and procedures that encourage staff to report such behaviors, is essential to dismantle the barriers created by psychological manipulation.

## Figures and Tables

**Table 1:** Demographic and work-related characteristics of nurses (*n* = 492).

Characteristics	N	%
Gender		
Males	88	17.9
Females	404	82.1
Age (years) ^a^	42.98	18.27
Years of work experience ^a^	9.79	9.95
Working in shifts		
No	90	18.3
Yes	402	81.7
Working in an understaffed department		
No	73	14.8
Yes	419	85.2

^a^ mean, standard deviation.

**Table 2:** Status of the study scales (*n* = 492).

Status of the Scales	N	%
Workplace gaslighting status		
Non-gaslightees	372	75.6
Gaslightees	120	24.4
Quiet quitting status		
Quiet quitters	272	55.3
Non-quit quitters	220	44.7

**Table 3:** Pearson’s correlation coefficients for the study scales (*n* = 492).

Scale	1.a	1.b	2	2.a	2.b	2.c
1. Gaslighting at Work Scale	0.938 **	0.968 **	0.305 **	0.221 **	0.300 **	0.198 **
1.a. Loss of self-trust		0.820 **	0.268 **	0.233 **	0.245 **	0.142 **
1.b. Abuse of power			0.308 **	0.195 **	0.317 **	0.224 **
2. Quiet Quitting Scale				0.822 **	0.836 **	0.695 **
2.a. Detachment					0.510 **	0.322 **
2.b. Lack of initiative						0.460 **
2.c. Lack of motivation						

** *p*-value < 0.01.

**Table 4:** Logistic regression models with perceived quality of care and patient safety as the dependent variables (*n* = 492).

Dependent Variable		Univariate Model			Multivariable Model ^a^		
	*Independent Variable*	Unadjusted OR	95% CI for OR	*p*-Value	Adjusted OR	95% CI for OR	*p*-Value
Perceived quality of care							
	*Workplace gaslighting*	0.650	0.527 to 0.803	<0.001	0.655	0.529 to 0.810	<0.001
Perceived patient safety							
	*Workplace gaslighting*	0.553	0.445 to 0.686	<0.001	0.561	0.450 to 0.700	<0.001

^a^ Multivariable models are adjusted for gender, years of work experience, working in shifts and working in an understaffed department. OR: odds ratio; CI: confidence interval.

**Table 5:** Linear regression models with quiet quitting as the dependent variable (*n* = 492).

	Univariate Models			Multivariable Model ^a^		
	Unadjusted Coefficient Beta	95% CI for Beta	*p*-Value	Adjusted Coefficient Beta	95% CI for Beta	*p*-Value
Workplace gaslighting	0.223	0.161 to 0.284	<0.001	0.224	0.163 to 0.285	<0.001

^a^ Multivariable model is adjusted for gender, years of work experience, working in shifts and working in an understaffed department. R^2^ for the multivariable model = 13%, *p*-value for ANOVA < 0.001. CI: confidence interval.

## Data Availability

The data used in this study are openly available in Figshare at https://doi.org/10.6084/m9.figshare.30972223.

## Supplementary Materials

The following supporting information can be downloaded at: https://www.mdpi.com/article/10.3390/healthcare14040450/s1, Figure S1. Histogram of the residuals with quiet quitting as the dependent variable; Figure S2. Scatterplot of residuals versus predicted values with quiet quitting as the dependent variable.
