# Supplementary figures and images for: Association Between Workplace Gaslighting and Perceived Quality of Care, Patient Safety and Quiet Quitting: A Cross-Sectional Study Among Nurses in Greece
Source: Healthcare (Basel). 2026 Feb 11;14(4):450. doi: 10.3390/healthcare14040450 (PMC12941031; doi:10.3390/healthcare14040450)

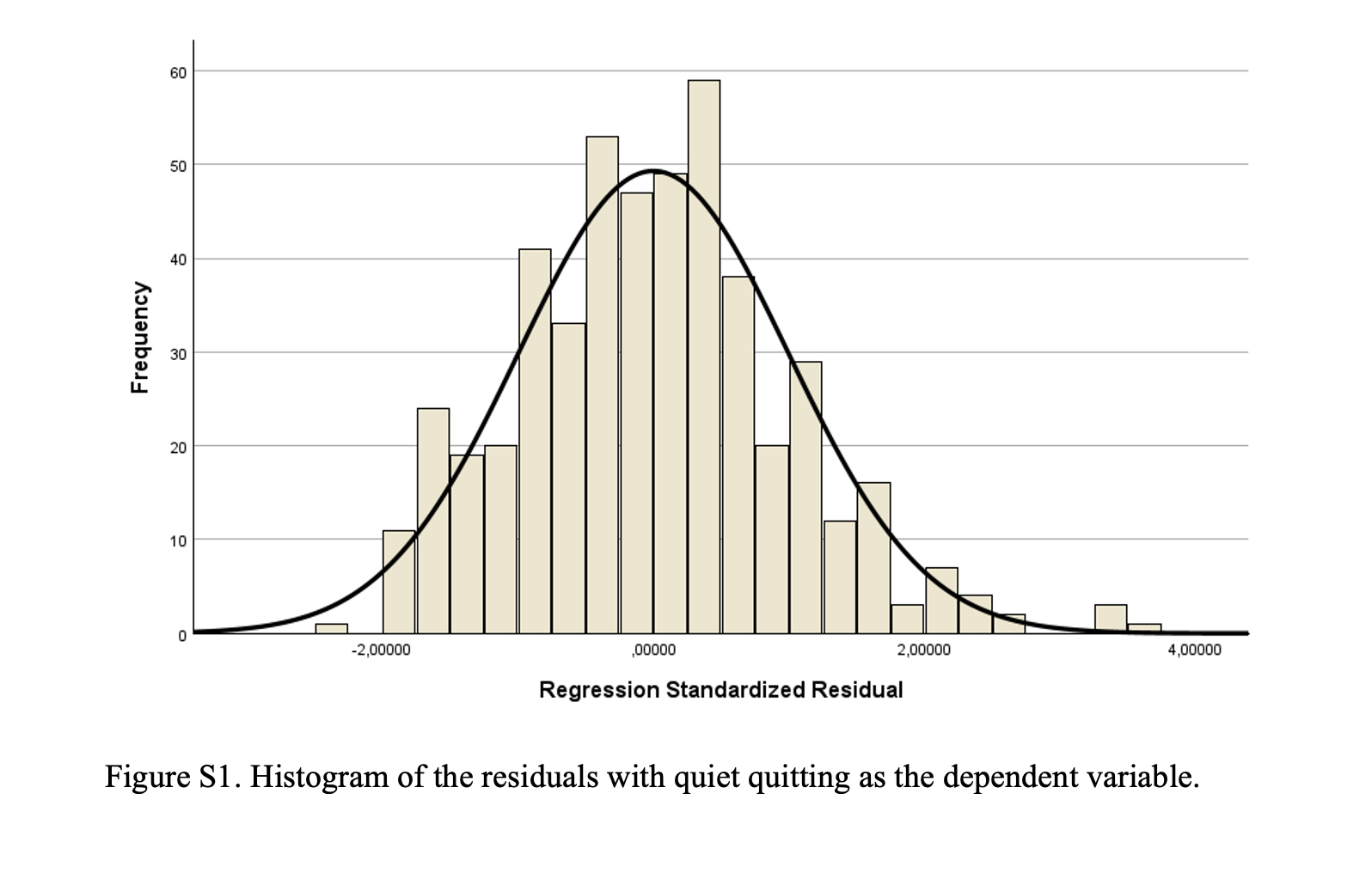

Supplement: Supplementary file 1 [file healthcare-14-00450-s001.zip › Figure S1.jpg]

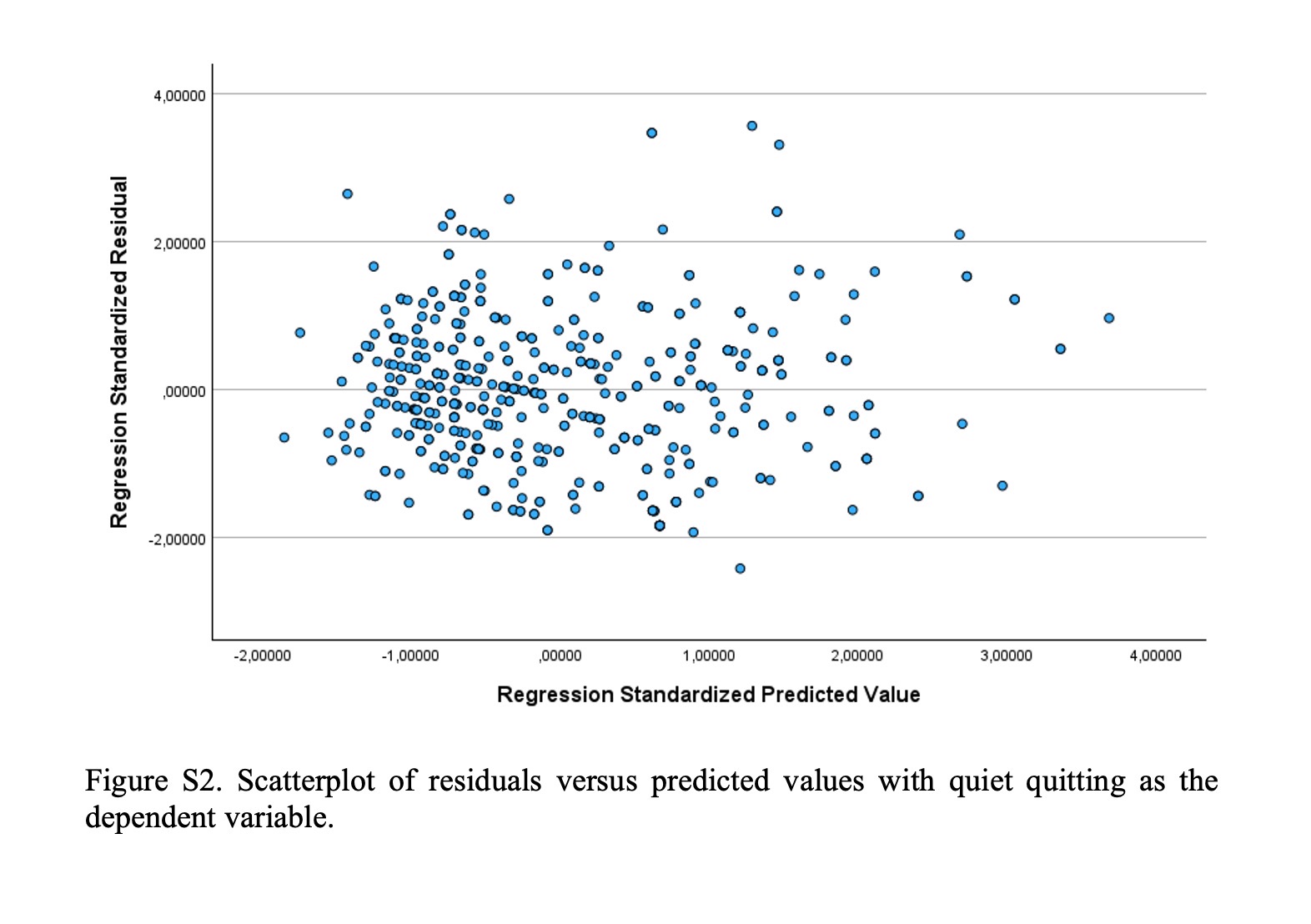

Supplement: Supplementary file 1 [file healthcare-14-00450-s001.zip › Figure S2.jpeg]
